# How Patient Work Changes Over Time for People With Multimorbid Type 2 Diabetes: Qualitative Study

**DOI:** 10.2196/25992

**Published:** 2021-07-15

**Authors:** Kathleen Yin, Joshua Jung, Enrico Coiera, Kenneth W K Ho, Sanjyot Vagholkar, Ann Blandford, Frances Rapport, Annie Y S Lau

**Affiliations:** 1 Centre for Health Informatics Australian Institute of Health Innovation Macquarie University North Ryde Australia; 2 Faculty of Medicine, Health, and Human Sciences Macquarie University Sydney Australia; 3 UCL Interaction Centre University College London London United Kingdom; 4 Centre for Healthcare Resilience and Implementation Science Australian Institute of Health Innovation Macquarie University Sydney Australia

**Keywords:** patient work, self-management, diabetes, chronic conditions, chronic illness trajectory, consumer informatics

## Abstract

**Background:**

The experiences of patients change throughout their illness trajectory and differ according to their medical history, but digital support tools are often designed for one specific moment in time and do not change with the patient as their health state changes. This presents a fragmented support pattern where patients have to move from one app to another as they move between health states, and some subpopulations of patients do not have their needs addressed at all.

**Objective:**

This study aims to investigate how patient work evolves over time for those living with type 2 diabetes mellitus and chronic multimorbidity, and explore the implications for digital support system design.

**Methods:**

In total, 26 patients with type 2 diabetes mellitus and chronic multimorbidity were recruited. Each interview was conducted twice, and interviews were transcribed and analyzed according to the Chronic Illness Trajectory Model.

**Results:**

Four unique illness trajectories were identified with different patient work goals and needs: living with stable chronic conditions involves patients seeking to make patient work as routinized and invisible as possible; dealing with cycles of acute or crisis episodes included heavily multimorbid patients who sought support with therapy adherence; responding to unstable changes described patients currently experiencing rapid health changes and increasing patient work intensity; and coming back from crisis focused on patients coping with a loss of normalcy.

**Conclusions:**

Patient work changes over time based on the experiences of the individual, and its timing and trajectory need to be considered when designing digital support interventions.

**International Registered Report Identifier (IRRID):**

RR2-10.1136/bmjopen-2018-022163

## Introduction

### Background and Significance

**Figure 1:**
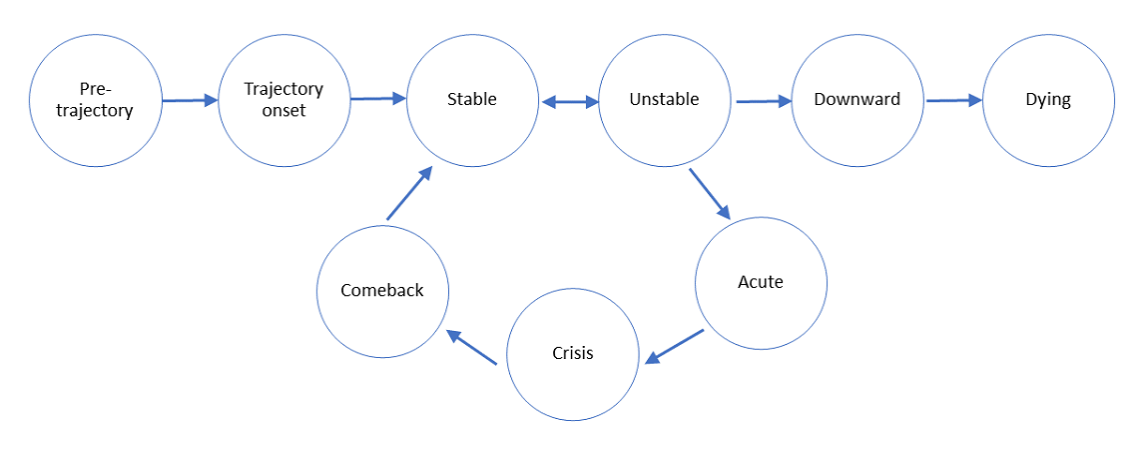
The Chronic Illness Trajectory Model.

Patient work is defined as health-related tasks and actions a patient undertakes in their self-management of health conditions [1,2]. Initially proposed by Corbin and Strauss [3], the concept recognizes that patients conduct a variety of such actions, with tasks ranging from physical to cognitive, ranging from visible to invisible, conducted alone, or requiring assistance from others [4]. The Chronic Illness Trajectory Model [5], also created by Corbin and Strauss, describes how the course of illness changes over time. Patients may shift between different illness phases repeatedly over their lifetime, as their conditions fluctuate (Figure 1 [5]). This model has been used to describe patients with conditions such as injury rehabilitation [6], metastatic cancer [7], poststroke recovery [8], and multiple sclerosis [9], with evidence suggesting that patient work needs and goals change as participants move between phases [6-8].

However, current digital technologies for patients with chronic disease struggle to incorporate changing health needs and goals. For example, newly diagnosed patients with type 2 diabetes prefer information on lifestyle alteration and available treatment [10,11], whereas patients with long-term complications, such as established diabetic retinopathy, require strict symptom monitoring [12,13]. Specific subpopulations have significant differences in health care preferences and goals, requiring varied information and recommendations. Studies that assessed targeted subpopulations have revealed that patients at different stages of their health condition have different information preferences [11] and that the use of apps is heavily influenced by contextual factors [14]. As such, patients experience *growing out* of the apps they took up early in their diagnosis, taking up new apps as they find their health care goals changing over the course of the illness.

Digital systems that are sensitive to the changing needs of their users would be able to help people keep using the same app over time, reducing the need to seek out more appropriate apps and ensuring continued tracking of health care data over time, especially for those who have comorbidities affecting multiple aspects of their health.

Therefore, gaps exist in our understanding of how patient work tasks and patient needs change over the phases of the chronic illness trajectory and how digital health apps can be improved by being designed with such changes in mind. We contribute to solving this issue by identifying the types of tasks patients engage in at different phases of their type 2 diabetes and the types of trajectories patients may experience over time and providing suggestions on how digital interventions could be designed to detect or anticipate changes in illness phases to provide maximum support.

### Objective

In this paper, we examine how patient work tasks and goals change over the chronic illness trajectory, focusing on multimorbid patients with type 2 diabetes mellitus self-managing in the community.

## Methods

### Overview

We undertook interviews of multimorbid community-dwelling patients with type 2 diabetes mellitus, as described in detail in the complete study protocol [15]. Ethics approval was obtained from the Macquarie University Human Research Ethics Committee for Medical Sciences (reference number 5201700718).

### Recruitment

Participants were recruited purposively from endocrinology clinics across metropolitan Sydney. Inclusion criteria were fluency in English, diagnosis of type 2 diabetes mellitus with at least one chronic comorbidity, and ability to legally provide consent.

Potential participants were suggested by clinicians, followed by researchers approaching these patients with a telephone call, explaining the purpose of the study and the processes involved. The researchers then sent a study information pamphlet and consent form via email or mail. If the person agreed to participate, the researcher arranged a time and location for the interviews. The researcher did not disclose their professional backgrounds.

In total, 26 participants were interviewed twice. A total of 52 individuals were approached during the recruitment. From the pool of 52 people, 5 (10%) did not meet the selection criteria and were excluded (2/52, 4% did not have comorbidities and 3/52, 6% did not speak English to the required standard), 6 (12%) agreed to participate but later withdrew, and 15 (29%) declined the invitation or were unable to participate.

### Data Collection

Each participant was interviewed on two occasions over 2 consecutive days, with each interview taking approximately 1 hour. Between interviews, participants wore a wearable camera for continuous, unobtrusive observation, as part of a larger study [15].

Interview questions were semistructured and focused on how community-dwelling people with chronic multimorbidity managed health-related tasks and how they modified their daily lives to accommodate their health needs. The interview question guide is provided in Multimedia Appendix 1 and was developed after consultation with the researchers. The interview questions and procedures were pilot tested with 2 participants before participant recruitment.

All participants were interviewed in their homes based on their choice. Many had arranged for family members to be present, with 38% (10/26) of participants having their spouses present, 4% (1/26) having their child present, 4% (1/26) having their mother present, and 4% (1/26) having their grandchild present. Family members often did not get involved and listened to the conversation. However, they were able to provide information when participants themselves were uncertain and asked for help.

All interviews were conducted by 2 researchers, one of whom was always KY (female), JJ (male), or AYSL (female). All interviews were audio recorded. Field notes were made by the interviewers during the interview to record nonverbal cues and observations inside the dwelling, and notes from both interviewers were consolidated within 24 hours of each interview. The second interview for each patient was conducted by the same researcher pair as in the first interview. The field notes were read alongside interview transcriptions, and any extra information was added to the end of the transcriptions.

### Qualitative Data Analysis

Audio recordings were transcribed externally and imported into NVivo Plus (QSR International, version 12). A thematic analysis was conducted inductively-deductively on all interview transcripts.

KY and JJ coded all transcripts separately and established codes that emerged from the data, which described either the work conducted to manage health or how the experience of self-management changed over time. These emerged codes were then deductively placed into themes that aligned with the phases and trajectories in the Chronic Illness Trajectory Model [5] and patient work tasks established in our previous scoping review [16]. Codes that did not fit into any existing themes in either of the frameworks were then read and placed into emergent themes over multiple readings. These new themes were presented alongside themes derived from the two frameworks.

Interview data were analyzed immediately after each interview to detect data saturation, and recruitment ceased when data saturation was reached (defined when no new patient work tasks were being described by 2 participants in a row). KY and JJ reviewed the codes and the theme framework over 6 months, and any discrepancies were resolved via consensus, with monthly meetings. KY, JJ, and AYSL then conducted design ideation [17-19], with brainstorming and scanning the available literature to consider the possible design implications of each need.

In this paper, we report our findings on patient work tasks and how they fit into the Chronic Illness Trajectory Model. Contextual factors influencing tasks and trajectories are beyond the scope of this study.

## Results

### Participant Demographics

The 26 participants of this study resided across metropolitan Sydney and had a variety of cultural backgrounds (Table 1). The median and mean age were both 72 years (range 46-86), with 61% (16/26) being male and 53% (14/26) identifying as Anglo Australian. The median and mean number of years diagnosed with type 2 diabetes were 19.5 (range 3-50) years. Less than two-third (16/26, 61%) of the participants were using insulin at the time of the study, and 69% (18/26) were retirees. The most common comorbidities were cardiovascular diseases, dyslipidemia, and kidney conditions, with a mean number of 3.96 comorbidities (range 1-20) per person.

**Table 1:** Participant demographics data (N=26).

Patient demographics			Participant, n (%)
**Gender**			
	Female	10 (38)	
	Male	16 (62)	
**Ethnicity**			
	Anglo Australian	14 (54)	
	Chinese	4 (15)	
	Indian	2 (8)	
	Italian	2 (8)	
	Trinidad and Tobago	1 (4)	
	UK migrant	1 (4)	
	Indonesian	1 (4)	
	Sri Lankan	1 (4)	
**Age (years)**			
	<60	2 (8)	
	60-64	3 (12)	
	65-69	3 (12)	
	70-74	6 (23)	
	75-79	7 (27)	
	80-84	2 (8)	
	85-89	3 (12)	
**Using insulin**			
	Yes	16 (62)	
	No	10 (38)	
**Major comorbidity (self-identified)**			
	Cardiovascular conditions	12 (46)	
	Dyslipidemia	3 (12)	
	Kidney conditions	3 (12)	
	Ocular conditions	2 (8)	
	Thyroid conditions	2 (8)	
	Prostate conditions	1 (4)	
	Mental health conditions	1 (4)	
	Osteoporosis	1 (4)	
	Traumatic injury	1 (4)	
**Duration of illness (years)**			
	<10	3 (12)	
	10-14	5 (19)	
	15-19	5 (19)	
	20-24	5 (19)	
	25-29	3 (12)	
	>29	5 (19)	
**Number of comorbidities**			
	1	4 (15)	
	2	9 (35)	
	3	1 (4)	
	4	4 (15)	
	5	3 (12)	
	6-10	4 (15)	
	>10	1 (4)	
**Employment**			
	Retired	18 (69)	
	Self-employed	3 (12)	
	Employed by others	5 (19)	

### Phases in the Chronic Illness Trajectory

Table 2 outlines the nine phases in the Chronic Illness Trajectory Model, their definitions [16], and example quotes from our cohort. Table 3 lists the patient work tasks involved in each phase. From our participants, we identified examples of patient work tasks in the following phases: trajectory onset, stable, unstable, acute, crisis, and comeback. More quotes supporting each of the phases can be found in Multimedia Appendix 2. Details of patient work tasks can be found in our previous review [16], which outlines the different task categories, how the categories were defined and created, and what examples were available for each category.

The pretrajectory phase represents the presymptomatic period before symptom presentation. We did not include this phase in our analysis or reporting.

**Table 2:** Phases within the chronic illness trajectory with definitions and examples.

Phase	Definition	Themes	Examples quotes
Pretrajectory	Before symptom presentation	N/A^a^	N/A
Trajectory onset	Initial symptom presentation and diagnosis	Participants respond to the new diagnosis by contacting health professionals and receiving new information.	“[The endocrinologists] give us a list of what to eat and what not to eat. But sometimes you do it, sometimes you don’t.” [P13, female, age 78 years]
Stable	Symptoms are under control and life activities continue within the limitations of the symptoms	Participants try to overcome inertia and find a new normal to discover what works for them.	“There’s a group online, about 200 people that have all done low-carb [diet], lost 100 pounds...and got their blood A1Cs right down. It seems to be the answer to me.” [P14, male, age 63 years]
Unstable	Symptoms start to get out of control and life activities are adjusted to cope with increasing health demands	Participants react to instability, taking up new tasks, new tools, and new information.	“I’m probably on about 14 [medications] at the moment, because I’ve just had to add two tablets too...when I had my bloods done for my endocrinologist, it came back and I’m very low on iron...he’s put me on iron tablets.” [P6, female, age 72 years]
Acute	Severe exacerbations of symptoms that require normal life activities to be paused	Participants rely on others to maintain basic functionality by prioritizing certain health needs over others.	“When I got told I’m going to be on dialysis, well I had a lot of trouble trying to accept that and kept avoiding it, until I was so sick I had to go on it.” [P11, male, age 76 years]
Crisis	A critical or life-threatening situation where urgent medical care is required	Participants cannot conduct self-management and can only react to crisis points.	“I was not allowed to eat anything. I was not allowed to even drink water, because there was a possibility for surgery at that time.” [P1, male age 67 years]
Comeback	Gradually return to an acceptable level of everyday life	Participants adopt to long-lasting changes and deal with mental distress during adjustment to a new normal.	“Just getting you out of bed and walking, just walking up the end of the corridor and back and that used to exhaust me. But once it’s all over and done with you feel fine. Two weeks of rehab.” [P17, male, age 70 years]
Downward	Consistent decline in health	N/A	N/A
Dying	Final days before death	N/A	N/A

^a^N/A: not applicable.

**Table 3:** Patient work tasks involved in each phase.

Tasks	Phase					
	Trajectory onset	Stable	Unstable	Acute	Crisis	Comeback
Planning		✓^a^	✓			
Proactive management of risks		✓	✓			✓
Deliberate distraction		✓				
Adapt to social values and expectations		✓				✓
Creating mental coping strategies	✓	✓	✓			✓
Learning about the disease	✓	✓	✓	✓		
Diet control	✓	✓	✓			
Taking treatment	✓	✓	✓	✓	✓	✓
Conduct exercise		✓				✓
Monitor signs and symptoms	✓	✓	✓			✓
Medication management		✓	✓			✓
Self-manage comorbidities		✓	✓			
Use and maintain assistive devices		✓	✓			✓
Do-it-yourself symptom management tools		✓	✓			
Alter the physical environment		✓				✓
Seek medical help	✓	✓	✓	✓	✓	✓
Ask for help from family and friends		✓	✓	✓	✓	✓
Hire professional help		✓	✓			✓
Consult complementary therapy		✓	✓			✓
Search for and attend patient support groups	✓	✓	✓			
Teach others about their health		✓				

^a^The patient work task is involved.

The trajectory onset phase represented when participants first became symptomatic and entered the health system. This phase primarily focused on patients with a gradual disease onset in the Chronic Illness Trajectory Model and was associated with tasks such as visiting doctors and trying to understand medical information. In the stable phase, participants spent the longest time and conducted the most patient work, with a large variety of tasks identified according to our previous publication [16] such as diet control, monitoring signs and symptoms, attending patient support groups, and planning for a new routine.

As health conditions worsen, participants may enter and exit the unstable phase repeatedly, with the goal of patient work being returning to the stable phase. Participants responded to changing health demands in the unstable phase, visiting medical professionals more frequently and starting to use support devices such as walking canes.

The acute and crisis phases represented severe and life-threatening illnesses, respectively. The participants were typically hospitalized during this period. They conducted no patient work, and health professionals oversaw their well-being. In the comeback phase, where recovery and rehabilitation occurred, patient work focused on transitioning back to the stable phase, with specific tasks such as rehabilitating exercises or coping with mental trauma. Under most circumstances, participants would not return to their previous levels of health after an acute, a crisis, or a comeback cycle. Some participants may experience this cycle repeatedly in their lives.

The final two stages of the model, the downward phase (an irreversible deterioration in health) and the dying phase (the last few days before death) were not observed in this study.

### Different Types of Chronic Illness Trajectories

#### Overview

**Figure 2:**
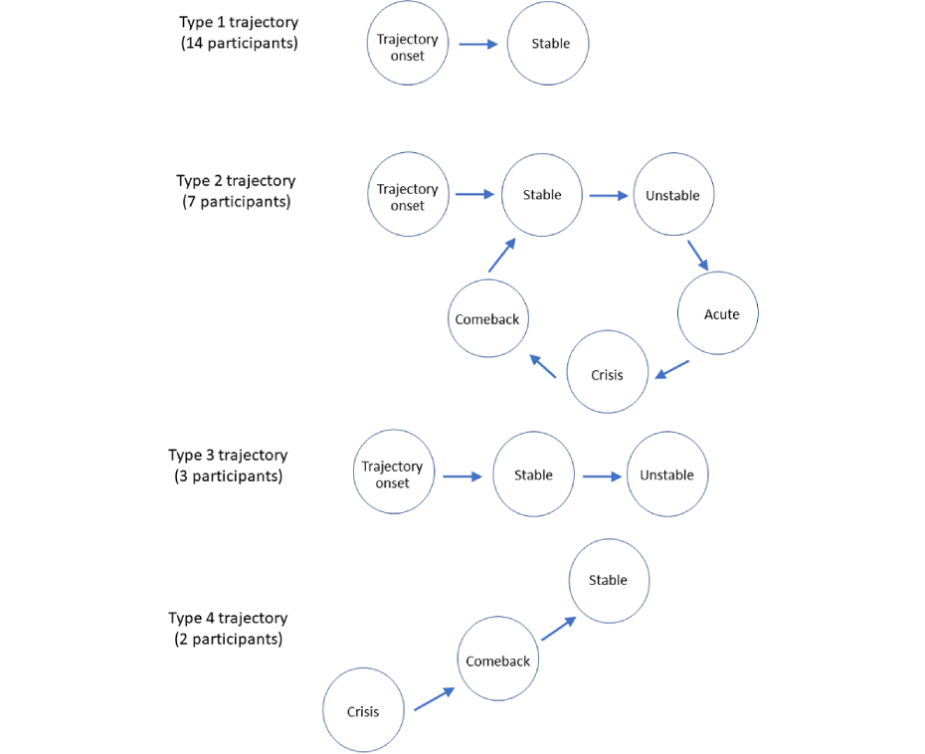
Visual representation of the four trajectories.

Participants experienced different patterns of change in their illnesses. Some remained stable for most of their disease trajectories, whereas others experienced many crisis episodes. We identified four unique trajectories in our cohort (Figure 2). Each trajectory represented a different life experience and required a different style of patient work adaptation. Each trajectory was also associated with unique goals and needs (Table 4). Multimedia Appendix 3 includes quotes supporting each trajectory. As participants are not at the end of the illness trajectory, it is possible that each participant could experience more than one trajectory in their lifetime. However, given that we are only able to capture past data and cannot predict future events, only the trajectory that each participant was experiencing at the time of the interview was reported here.

**Table 4:** Trajectory types and their characteristics (N=26).

Trajectory type	Explanation	Work goals	Participant IDs	Number of comorbidities, mean (SD)
Living with stable chronic conditions	Trajectory onset→stable (participants were diagnosed at a mild stage of the disease where conditions were stable and did not experience disease exacerbations).	Making patient work as routinized and invisible as possible.	7, 9, 10, 14, 15, 19, 20, 22, 23, 25, and 26	3.2 (1.7)
Dealing with cycles of acute or crisis episodes	Trajectory onset→stable→unstable→acute→crisis→comeback→stable (participants have experienced episodes of disease exacerbation, sometimes repeatedly).	Heavily multimorbid, experiencing polypharmacy, and requiring support with self-management adherence.	2, 3, 4, 8, 11, 12, 13, 17, 18, and 21	3.4 (1.7)
Responding to unstable changes in their conditions	Trajectory onset→stable→unstable (participants were diagnosed at a mild stage but are currently experiencing a decline in health).	Experiencing increasing intensity and variety of patient work and dealing with rapid changes.	6, 16, and 24	8.7 (9.7)
Coming back from crisis before stabilizing	Crisis→comeback→stable (participants were diagnosed due to a sudden and severe exacerbation and recovered from that crisis).	Cope with a total loss of normal life and needing holistic support.	1 and 5	1.5 (0.7)

#### Type 1: Living With Stable Chronic Conditions

The trajectory type most common in our cohort was for those participants who experienced many years of stability with their disease (14/26, 54% of participants). These participants had only experienced the trajectory onset and stable phases (Figure 2) without disease exacerbation. Their work goal was to maximize the integration of patient work into daily life, *making patient work as routinized and invisible as possible*.

Participants in this trajectory type were diagnosed at a mild stage of the disease. They had sufficient opportunity to experiment with patient work tasks, having tried different food options, exercise routines, medications, or information sources, for example. As this group had spent many years in the stable phase, these participants regarded patient work as an incorporated part of their lives. Some participants reported that their patient work had become so ingrained, they no longer remembered each medication’s purpose or the roles of the health practitioner they were seeing. Participants averaged 3.2 (SD 3.8) comorbidities in this trajectory, indicating a relatively mild stage of health deterioration.

#### Type 2: Dealing With Cycles of Acute or Crisis Episodes

The second most common trajectory type was observed in participants who experienced at least one incidence of acute or critical exacerbation followed by the comeback phase (7/26, 27% of participants). Apart from the phases experienced by participants in the type 1 trajectory, the type 2 trajectory also included experiences in the unstable, acute, crisis, and comeback phases (Figure 2). The work goal for this group revolved around being *heavily multimorbid, experiencing polypharmacy, and requiring support with self-management adherence.*

This type of trajectory described participants who had major critical episodes, such as strokes or heart attacks, at least once. Some participants experienced multiple critical episodes and accumulated more medications and diagnoses. Interestingly, due to their complicated medical histories, many participants in this trajectory type had an excellent understanding of medical information. They were aware of the biochemical principles behind their disease and medications but required managerial support to follow their complicated patient work routines. Participants averaged 3.4 (SD 1.7) comorbidities in this trajectory, suggesting a similar level of well-being to the type 1 trajectory group.

#### Type 3: Responding to Unstable Changes in Their Conditions

The third trajectory type included participants currently in the unstable phase (3/26, 12% of participants). These participants would eventually either proceed onto the acute, crisis, or comeback cycle, or recover enough to return to the stable phase. However, at the time when the interviews were conducted, this group experienced fluctuating health states and had specific work goals and user needs (Figure 2). Their patient work goals showed *increasing intensity and variety of patient work and dealing with rapid changes.*

Owing to their rapidly changing health, participants in this trajectory were seeing their doctors nonroutinely and frequently, with many changes being made to their treatment regimen in a short timeframe. These participants reported confusion regarding the purpose of their treatment and struggled to keep up with their health, often having to rapidly adopt assistive devices (eg, walking canes) or external helpers (eg, hired cleaners). These patients felt that their health was taking over all other spheres of life and required help with understanding how their health was progressing. Participants averaged 8.7 comorbidities (SD 9.7) in this trajectory, significantly higher than the number of comorbidities experienced by the previous two types and suggesting a worse stage of health.

As these participants are currently experiencing exacerbation in health, it is impossible to determine which phase and trajectory they will end up in. For most participants experiencing this trajectory, it is the first time they experience exacerbation, thus lacking the experience and familiarity that participants from the type 2 trajectory may have derived from previous experiences with worsening health. Therefore, we made a distinction for this group of participants, acknowledging the difficulties and stress associated with responding to unstable changes in their health.

#### Type 4: Coming Back From Crisis Before Stabilizing

The least common trajectory type in our cohort was the participants who received their diagnosis during a crisis event (2/26, 8% of participants). Unlike the other three trajectories, this group initiated their trajectory during the crisis phase (Figure 2), such as with a heart attack or a traffic accident. Their patient work goals focused on *coping with a total loss of normal life and needing holistic support.*

During the crisis phase, the participants were hospitalized for prolonged periods with little to no autonomy. As participants slowly recovered in the comeback phase, they reported experiencing great psychological trauma as they came to understand the irreparable changes in their health. Some participants indicated that they were incapable of coping with these changes during the comeback phase and experienced a range of negative emotions such as dread, devastation, powerlessness, or denial. Suicidal ideation, depression, and thoughts about death were explicitly mentioned. Participants averaged 1.5 comorbidities (SD 0.7) in this trajectory, the lowest number of all types, due to their recent diagnosis and being situated at a relatively earlier stage of the journey of illness despite having experienced significant trauma.

This category of participants did not strictly adhere to the chronic illness trajectory, as participants were not diagnosed at a mild stage of the disease. They also differed from the type 2 trajectory as they did not experience living with their condition in a controlled manner before symptoms worsened and began to go out of control. They do not have the knowledge taught to them by clinicians at diagnosis during the trajectory onset phase and had to learn about self-management while coping with trauma and recovery. Therefore, they have been given a distinct category to reflect their lack of familiarity with this newly diagnosed condition and the few resources compared with those of the other three trajectories previously discussed.

## Discussion

### Principal Findings

Determining the support needs of multimorbid patients self-managing in the community is an important initial step in designing digital interventions for them. Chronic comorbidities are extremely common in the type 2 diabetes population, with studies indicating that up to 97.5% of patients have one comorbid condition and 88.5% have at least two [20,21]. Our participants were also predominantly affected by comorbidities considered to be concordant with the pathophysiology of type 2 diabetes [22], such as cardiovascular diseases, dyslipidemia, kidney conditions, and ocular conditions. This group represents a population in the community burdened with significant patient work and poses major financial challenges to the health care system if not well-managed. Currently, health apps for chronic patients in the community either target broad populations, such as patients with heart failure, or narrowly defined subgroups, such as patients recently discharged following total knee replacement [23]. Our findings indicate chronic multimorbid patients, such as those with type 2 diabetes, have support needs that evolve over time and are much more complex than those currently supported. As such, a generic app for a certain disease cannot realistically support the differing needs of all patients irrespective of the phase or trajectory type they are in.

Our data revealed four distinct trajectory types over time that produced different self-management goals and work goals, further dissecting chronic multimorbid patients into subgroups based on their previous medical experience. Designing for patient needs from a viewpoint that includes previous medical history and current state is therefore likely to improve user acceptability and appropriateness of digital health apps, enabling such digital tools to provide more timely, suitable, and actionable advice.

### Designing for Phase-Specific Needs

Our study indicates that the range and intensity of patient work varies at different phases of the Chronic Illness Trajectory Model. Digital interventions that seek to optimize self-management should therefore be designed according to the specific needs of each phase and acknowledge that different types of support are required for different tasks in each phase.

Studies have begun to address this, moving away from generic *all-patient* user groups, with recent studies designing specific digital tools for newly diagnosed people with diabetes [24] and for people recovering from trauma [25], for example. Such studies have already uncovered significant differences in the subpopulations [11]. Patients using apps also report that as their self-management behaviors change, they can *outgrow* the apps that helped them early in their illness trajectory [26], resulting in continuously seeking out new apps. In our data, patients described different tasks and needs at various phases of the chronic illness trajectory. In the trajectory onset phase, patients commonly thought that the medical information provided was too generic, too technical, and did not translate into actionable suggestions in their own lives. This can be supported by giving personally relevant, precise, and clear advice (such as *eat less bread* and *walk up and down the stairs during lunch break* instead of *eat less carbohydrates* or *do more exercise*). During the unstable phase, patients were compelled to take risk management measures in aspects of life previously taken for granted, such as taping carpets to the floor to prevent slipping or putting rubber bands around stair banisters to feel the stairs at night. Patients in this phase need more information about what is happening to them and what they need to look out for on a daily basis, provided in lay language. The acute and crisis phases produced significant restrictions and burden to patients and their families, with participants describing having to conduct all activities on the mandate of doctors and feeling *shattered* or having *a lot of trouble trying to accept it*. Patients in these phases need mental health support and clear and actionable advice to reduce any chance of confusion or mismanagement. The comeback phase was described as a new lifestyle, with major adjustments to life needed to accommodate changes in routines, such as rehabilitation schedules or dialysis. Patients need logistical support at this stage, such as finding out how to obtain a wheelchair or arrange for subsidized transport, to cope with their reduced health state.

### Designing for Trajectories

The four types of trajectories identified in this study correspond to four distinctive design patterns. Each design needs to be tailored to the intended user’s needs, digital literacy, environment, and whether the user is the patient or their caregiver. On the basis of the work goals identified in Table 4 and Multimedia Appendix 3, we present the digital needs of the four trajectories in Table 5, together with potential tools that can address their needs. Patients may also shift from one trajectory type to another as their health changes over time, with their need for digital technology changing accordingly.

**Table 5:** Digital user needs and recommendations for each trajectory type.

Trajectory types	User needs	Potential digital tools
Type 1: living with stable chronic conditions	Tools that normalize patient work and remove the burden of having to think about the disease	Background data collection tools that require no user input (eg, step-counting phone apps) [27] Integrated, predetermined lifestyle changes and health intervention delivered automatically (eg, smart fridges that order specific groceries based on existing algorithms and automated prescription refill and delivery) [28]
Type 2: dealing with cycles of acute or crisis episodes	Tools that support self-management adherence and monitors health	Medication adherence support tools (eg, context-aware digital reminders that cue for medication taking immediately before meals) Crisis prevention technologies (eg, health monitoring tools that use predictive algorithms to observe signs, such as food and medication consumption, and generate alarms based on behavioral changes)
Type 3: responding to unstable changes in their conditions	Tools that provide symptom monitoring and give alarms for health deterioration	Crisis prevention technologies (eg, health monitoring tools that use predictive algorithms to observe signs, such as changes in physical symptoms and emotional states, and generate alarms based on symptom changes) Scheduling and communication assistance (eg, apps that can manage a complicated and changing timetable involving multiple clinicians)
Type 4: coming back from crisis before stabilizing	Tools that support coping with a total loss of normal life and guide patients toward appropriate services and support infrastructure to re-establish normalcy	Guide the patient to seek appropriate social services (eg, direct patients to appropriate social, financial, and legal services) Provide support with mental health and coping (eg, phone-based mental health support apps)

### Designing for Phase Change

Digital tools that aim to be used throughout the duration of a disease’s chronic illness trajectory will need to detect phase changes, such as when the patient’s health worsens from the stable to the unstable phase. Although collaborative, co-design exercises with patients to gain insight into user needs are now common during digital health app development, the circumstances of the patients’ health do not remain immutable after the app’s release. Tools to capture patient-reported experience measure and patient-reported outcome measure [29,30] can collect self-reported data at preset points of the day or immediately after predefined trigger events and can assist with keeping up to date with the patient’s health after the app’s release. Ideally, an app that detects phase changes should alter its functionality accordingly by activating submodules. For example, participants interviewed for an app designed for mental health expressed a desire for the app to send them mood-regulating messages at times when they were about to lose their temper [31]. This can be achieved either through purely automatic detection of worsening biophysical signs (such as constantly elevated blood pressure or lopsided gait) via external sensors, regular self-reporting by the patient, wearable smart household items such as smart mattresses or smart watches or using individual user data as baselines to train algorithms. Although such data can be entered into digital devices by the patient, automated data collection would reduce the health-related burden of self-monitoring. Excessive requirements for self-reported data could make digital interventions burdensome and contribute to disengagement and dropout over time. Other innovative, context-sensitive digital health interventions, such as *Smart Pill Bottles* that can detect irregularities in medication consumption [32,33], also have the potential to be integrated into a home system that generates external data complementing phone-based apps.

When the interviews took place, each participant’s trajectory described the person’s current state and journey to this point from the onset of their health conditions, with the future states of each person not necessarily known. Should future research discover these phases and trajectory types to be predictable based on medical history, digital tools can be designed to anticipate such changes and variations between different patients. Individualized and customizable apps [34,35] allow for further tailoring to fit specific subpopulations, and designs of self-care tools would need to adapt to the patient’s evolving digital needs to ensure relevance and integration into patient work.

For full realization and evaluation of any of the design implications suggested in this section, co-design sessions would have to be conducted with participants living in the targeted chronic condition, which is beyond the scope of this paper. However, the design implications highlighted here can serve to trigger discussion toward more innovative, holistic, and responsive digital intervention design during co-design sessions, particularly as very few digital interventions currently adapt to changing needs and trajectories.

### Limitations

This study was limited in terms of data collection. First, we only recruited participants with a clinical diagnosis who were not severely ill and asked them to recall patient work from the time of diagnosis rather than recruiting individuals at different phases of the chronic illness trajectory. This was done to understand how patient work has evolved for each person. Second, our sample included more males than females because more females declined participation due to family concerns or obligations. Finally, our recruitment criteria stated that participants must be fluent in English. Consequently, the patient work of people who were not fluent in English was not captured.

### Conclusions

This study provides insights into how patient work among multimorbid patients with type 2 diabetes changes over time. There are still gaps in our understanding of how patient health care goals change through different phases of their health, how different patients have different disease trajectories, and how digital health apps can adjust to such changes over time. This study presents data on different types of trajectories, with the perspective of how to use such findings to design better consumer-facing digital health apps. Our findings revealed four different types of trajectories, resulting in different patient work goals. Patients who had never experienced disease exacerbation desired for patient work to be as invisible as possible, whereas those who lived through cycles of crises and recovery needed assistance with self-management adherence. Participants currently experiencing a decline in health needed timely support and crisis prevention technology, and those diagnosed during severe crisis needed guidance to find sources of support and coping. This study highlights opportunities for health informatics and design communities to explore the untapped space of designing for time and trajectory, where future research should incorporate an individual’s evolving health experiences when designing digital technologies for patient work over time.

## Appendix

Multimedia Appendix 1 Interview question guide.

Multimedia Appendix 2 Quote table for different phases.

Multimedia Appendix 3 Quote table for different trajectories.

## Abbreviations

NHMRC: National Health and Medical Research Council
