# Supplementary material for: How Patient Work Changes Over Time for People With Multimorbid Type 2 Diabetes: Qualitative Study
Source: J Med Internet Res. 2021 Jul 15;23(7):e25992. doi: 10.2196/25992 (PMC8323019; doi:10.2196/25992)
Supplement: Multimedia Appendix 1 [file jmir_v23i7e25992_app1.doc]

**APPENDIX 1 – INTERVIEW QUESTION GUIDE**

**Interview 1:**

Physical health and medical interactions:

- What health conditions do you have?
- What medications do you take?
- What kinds of health professionals do you see?
- What are your opinions about your medications and your doctors?
- Do you see any complementary or natural health practitioners? Why?
- What has been your experience with your health conditions?

Physical environment:

- Did you ever buy assistance equipment, or modify your house in any way, to accommodate your health?
- How do you find driving and moving around in general?
- How have you ‘re-arranged’ your house to accommodate your health?
- Has the physical layout of your house or any other place affected how you managed your health?

Self-management tasks:

- Do you exercise? If so, what do you do, and how often?
- How do you feel about your exercise routine?
- What do you usually eat for breakfast, lunch, and dinner?
- What kind of food do you need to watch out for?
- How do you manage to obtain and cook the right kind of food for yourself?
- How does your diet requirement make you feel?
- Do you monitor your own symptoms? How often do you do it?
- Why do you monitor your symptoms at this specific frequency?

Mental burden and mental coping:

- Can you tell me about what you felt when you were first diagnosed and/or when you first started medication?
- How did your health condition and having to take the treatment make you feel?
- Do you feel differently towards your health conditions compared to at the start?
- Do you feel depressed or anxious because of your health?
- How have you managed those anxieties?

Organisational management:

- Has your family, work, or hobbies been affected due to your health?
- How have you ‘re-arranged’ your life to accommodate your health?
- How have you incorporated your treatments into your daily routine?
- Have you had to make changes regarding your work because of your health?
- What do you do with your medications when you travel?
- How do you keep track of appointments and medicines?

Health knowledge:

- Do you know which of your medications are for what condition?
- Do you understand the biology behind your conditions?
- How did you learn about your health conditions and your treatments?
- What do you feel about the process of obtaining this information?

External help:

- Do you get any domestic help with cleaning, cooking, gardening, or any other chores?
- Do you have family or friends helping out?
- Are you a part of any patient support groups?
- Do you help out anyone else with your health conditions?

**Interview 2 (during photo viewing):**

- How was your experience with the camera?
- How was your day?
- Was it a typical day? If not, what was unusual?
- How did you sleep during the study night?
- Do you always do [activity] at this time of the day?
- Is this the kind of food you usually eat for breakfast/lunch/dinner?
- You spent a long time doing [activity] yesterday. Is this how long you usually take?
- Do you always stay at [location] around this time?
