# Supplementary material for: How Patient Work Changes Over Time for People With Multimorbid Type 2 Diabetes: Qualitative Study
Source: J Med Internet Res. 2021 Jul 15;23(7):e25992. doi: 10.2196/25992 (PMC8323019; doi:10.2196/25992)
Supplement: Multimedia Appendix 2 [file jmir_v23i7e25992_app2.doc]

| **Phase** | **Examples quotes** |
| --- | --- |
| Pre-Trajectory | N/A |
| Trajectory Onset | *They [the endocrinologists] give us a list of what to eat and what not to eat. But sometimes you do it, sometimes you don't. (P13, female, age 78)*  *The first thing that happened was they sent me off to - and I don't even know if they do them anymore, but a diabetes educator… So they gave me a little bit of information about what's happening with the pancreas, the technical stuff (P26, female, age 46)* |
| Stable | *There's a group online, about 200 people that have all done low-carb [diet], lost 100 pounds… and got their blood A1Cs right down. It seems to be the answer to me. (P14, male, age 63)*  *I got a little strip that I put under my mattress now called Beddit and that measures your respiration and heart rate and everything. It's helping me get more sleep. (P14, male, age 63)*  *On a certain day - in fact it happens to be Tuesday. That's today. I get out all the pills and put them in there. (P16, male, age 78)*  *NovoRapid, although it did stabilise my diabetes, it was a - that's was why I had to know so much about what medication I take. Because I had to rely on my blood sugar reading to determine how much NovoRapid I'd have. I'd have NovoRapid in the morning and the night. But if I was I was going out to have a big lunch then I used to substitute for the lunchtime, and only a very small dose at night. I'd get low BGL readings. (P8, female, age 85)*  *I check my blood sugar in the morning and, depending on what that is, I have a small bowl of cereal. Then if my blood sugars are low - and after the cereal, I take about six units of insulin. Then I go and have lunch. Then two hours after lunch, I check it again and if I'm in the range, I don't worry too much. I can't take any more insulin until the evening. But I know that I've taken enough in the morning just to see me through the day. (P11, male, age 76)*  *We've had the education with the diet with the diabetes crowd. So I know what to eat, or I do most of the time, yeah. (P2, male, age 81)*  *If I want to know, I'll write down if I want to something. When I go to the doctor I'll say look, I heard on television or radio this new thing blah blah that's happening. Now what's your opinion of it? How do you feel about it? I don't know if they're happy explaining it to me (P8, female, age 85)*  *For education or new technique, I have to know. Like I've got diabetes I want to know everything. (P23, female, age 71)*  *You mentioned you see a dentist and an eye doctor, you see them once a year, these two?*  *Male: Probably twice a year.*  *Facilitator 1: Twice.*  *Facilitator 1: Mm, and then the heart specialist once a year.*  *Male: Once a year.*  *Facilitator 1: The skin specialist every six months.*  *Male: Six months, yeah. (P2, male, age 81)* |
| Unstable | *I'm probably on about 14 [medications] at the moment, because I've just had to add two tablets too, because my - when I had my bloods done for my endocrinologist, it came back and I'm very low on iron… I went to my GP and he's put me on iron tablets. (P6, female, age 72)*  *All my glasses were useless except the ones for reading. So I had to go back and get another prescription. That lasted not very long. I've got to go back and get another one, but I don't know where I'm going… I'm not going back to Specsavers. They just couldn't get [it] right. (P17, male, age 70)*  *I used to go every week but since last year I've stopped and only been out a few times. Because when I came back I found that I feel distressed about my body health. So that's why I haven't been out as soon as I, as I like to. But no, in the last few months the health is really, really bad. So I just stop all together. (P24, male, age 75)*  *Every time you start on a new medication - like this is a new one that I will start that on Monday night, I don't know how yet I'm going to react to that. So it's always that back of your mind, okay, I've got to - you know, everything's working fine, now we're changing one of the meds, how is that going to work, how is it going to interact with all the others? So there's always that back-of-your-mind concern. (P26, female, age 46)* |
| Acute | *When I got told I'm going to be on dialysis, well I had a lot of trouble trying to accept that and kept avoiding it, until I was so sick I had to go on it. (P11, male, age 76)*  *[During] the hypos I get sick and I get dizzy, I can't see and, you know. If it happens in the middle of the night it's very hard because you can't even hardly talk, you've got to get up and you've got to find some sugar somewhere. So I come out here and I guts down as much sugar as I can throw in my face. (P17, male, age 70)*  *… the first time I had to inject myself with the insulin and I physically couldn't do it. I just couldn't do it. I was so upset, I was just inconsolable and luckily my husband is also a diabetic, so I was like, you have to do it for me… I was shattered. I was just absolutely gutted by it. (P26, female, aged 46)*  *As soon as there is something happening and I start to feel shaky, I just stop everything and go and get something to eat. So I won't let me have that problem. Straight away I know. I start shaking and I think hang on. (P19, male, age 65)*  *I think I'll be in intensive care for a week and then they like to send you home after that. I'd say it'd be intensive care for a week, another week just monitoring (P4, male, age 48)* |
| Crisis | *I was not allowed to eat anything. I was not allowed to even drink water, because there was a possibility for surgery at that time. They didn't want any [food in the stomach]… He [the surgeon] said, you just can have one cube of ice now and then, that's it (P1, male age 67)*  *When I couldn't breathe, I used it and I didn't get any reaction, so I got my husband to rush me up to the hospital. (P8, female, age 85)*  *He [the doctor] said I've got to give you bad news. You've got a blockage in the main artery, 97 per cent blocked. I said, oh yeah, I'm lucky I've still got 3 per cent working. He said, please don't. He said, you can't move. You can't get up. If you want to go to the toilet go to the people to bring you a pan or whatever… but you're not allowed to move. (P12, male, aged 79)* |
| Comeback | *It was mainly walking. Just getting you out of bed and walking, just walking up the end of the corridor and back and that used to exhaust me. But once it's all over and done with you feel fine. Two weeks of rehab. (P17, male, age 70)*  *I'm not trying to tell lies, all sorts of thoughts went through my mind… I walked from here to [train] Station one day, which is over three kilometres. It's quite a distance. Sat on the railway station hoping to jump in front of a train that's how close I got. (P1, male, age 67)*  *It [dialysis] is a new lifestyle, mm. Five hours three days a week is not fun… Yeah, my time slot's six am in the morning, so I get in there 5:30am. So I've still got most of the day when I get out. (P11, male, age 76)*  *This afternoon I have to go and get that stick that you can use for scratching your back, because you can't - I just cannot reach that part of my back. (P1, male, age 67)* |
| Downward | N/A |
| Dying | N/A |
