# Supplementary material for: How Patient Work Changes Over Time for People With Multimorbid Type 2 Diabetes: Qualitative Study
Source: J Med Internet Res. 2021 Jul 15;23(7):e25992. doi: 10.2196/25992 (PMC8323019; doi:10.2196/25992)
Supplement: Multimedia Appendix 3 [file jmir_v23i7e25992_app3.doc]

| Trajectory type | Example quote |
| --- | --- |
| 1. Living with stable chronic conditions | *As I say, I don't know what I take them for, I just line them up and take them. (P7)*  *When you start preparing everything, you will enjoy the life. (P8)*  *you only sort of half-believed you had diabetes, because there were no real side effects that you could see, and you didn't feel bad or anything. (P14)*  *managing my condition has made it a bit - well just you've got to stop and take your bloods and things like that so you've got to think about that (P14)*  *I just get - you feel a bit frustrated that you can't seem to get past it at times. It's always in the back of your mind, but you sort of cope. (P15)*  *the only thing that'd differ is if the warfarin changes, like you have the INR today and it's too low or too high I might have to break that tablet in half or take a two milligram and a three milligram or a two milligram and a one milligram rather than five milligram, or take a one or a two on top of that as well. It just depends. (P17)*  *I still walk past a cake shop like this [avert eyes], I don't look, because I can't stand looking at the [desserts]. (P17)*  *After I was diagnosed with diabetes I became instantaneous food fanatic, I stopped everything and I only ate good stuff. But then it finally crept back in and I had a this or that. You just can't, you can't stay away from it after you've been living with it for as long as I was and then to tend to have a, have to stop it. I went off, I didn't drink any Coke whatsoever, didn't drink any fizzy drinks, just water. But that crept back in again, too. (P17)*  *It's very difficult sometimes to understand that your life can't be the same. Your sex drive is completely compromised because diabetes affects the nerves and affects the peripheral nerves. So you have erectile dysfunction, that's a big major problem and you just can't get erections, so sex is something that just doesn't happen. You have problems with feeling, touching things and getting a sensation from it. (P17)*  *I'm not a brilliant manager. I need to be reminded of things by my wife as I mentioned. I don't think I'm as concerned about my health issues as what she is (P17)*  *because of the diabetes, I know that you've got to exercise. You've got to take the tablet. That's become part of my life. You have something to eat, the medicine, and then go out. (P19)*  *it will happen, because that's the fact. Diabetes is a chronic disease, so it's not going to go away. The only thing - I know that diabetes, managing it, you can't get rid of it. There's no cure. So that's why sometimes yes, sometimes I worry what's going wrong, that sort of thing. (P19)*  *that’s mean I have to take this for the rest of my life. Not allowed to forget once (P23)*  *I’ve got two [medicine packs]. So that way, I’ve covered myself because I’m afraid to forget. Even that, lately, so far, the last five years, twice I forget. I thought, gee, if I got out of routine, and the next thing I forget. (P23)*  *So there's lots of different things. So it is constantly on your mind with the diabetes particularly, but they are probably the two most difficult conditions to manage, the diabetes and the variety (P26)*  *I try to co-ordinate it if I can. So I've set up early appointments. So I try to set up appointments a long time in advance. So I've already got a session booked with the endocrinologist, the exercise physiologist and the dietician in one block for February of next year. So if you book it that far in advance, you can get the times together, but for this round I was there yesterday, I'll be there next week. It's a lot. So it's a lot of running around. (P26)*  *I've got it on an app now which is really convenient. So, I just look at it and it actually tells me when I'm about to run out of the medication which is fantastic. It's made it a lot easier (P26)*  *it used to be a case of you take your medication at night and you go, oh, no, I've got two tablets left. I don't know if the script is at the pharmacy or if it's here. So you have go rummaging for it and I don't know about you but I'm not the world's most organised person. So I'd rummage for the script and then I couldn't find it. Then I'd have to ring the pharmacy and they didn't have it and then you'd be like, oh my god, now I've got to go back to the GP and get another. It was a nightmare, especially when you're taking that many medications. (P26)*  *there's no holistic treatment and I think that's really to their detriment. That's fine if you're a really healthy person who just occasionally needs a script for a cold or something, but if you've got more complex needs you really have to have almost like a case manager. (P26)*  *the last one I had was Bydureon. You basically just take that out half an hour before you're ready to inject it and the rest of the time it just stays in the fridge. So this one I find a little bit tricky to remember because I only take it once a week. So I've just got an alarm set up on my phone to remind me to take it. (P26)*  *I don't even worry about them anymore. Like even the sleep apnoea, I've got a machine for that, so I control that, I don't even think about it anymore. Whereas the other stuff is still like part of my everyday life. You know, I've got to take the medication, I've got to take the injections, I've got to do all that sort of stuff. So from that perspective, yeah, there's definitely some that are more serious than others. (P26)*  *Every time you start on a new medication - like this is a new one that I will start that on Monday night, I don't know how yet I'm going to react to that. So it's always that back of your mind, okay, I've got to - you know, everything's working fine, now we're changing one of the meds, how is that going to work, how is it going to interact with all the others? So there's always that back-of-your-mind concern. (P26)*  *It's one o'clock… crap, I don't know what to have [for food]. Then you go through the thought processes associated with that of oh, god what am I going to have? What can I have that's healthy? What can I have that's not going to shoot the blood sugar levels up? It's never just a case of I'll grab a sandwich. It's never ever that easy. There is always a lot of ruminating in the head first about what am I going to have? How many units of insulin will I have to have to counter that? Am I doing any exercise this afternoon? (P26)* |
| 1. Dealing with cycles of acute or crisis episodes | *Now, with these, I was only taking one a day, but I'm now taking two, because [doctor]’s upped it to five mg. So when I get the new script, it'll just be back to one tablet a day, but it'll be five mg, because you can see that's, I think, 250 [micrograms], isn't it? Yeah. One of those a day. Just don't confuse it. That's basically it. (P4)*  *Tritace can interfere with your kidneys and I was hospital. I had a complete shutdown of kidneys, pancreas, liver, everything. So they had to cut back the Tritace because it does affect your liver. (P8)*  *If they put me on a new tablet I want to know why and what it's for, and what will be the side effects of it. (P8)*  *It's [dialysis] a new lifestyle, mm. Five hours three days a week is not fun (P11)*  *it's [wearable alarm] all automatic. If I'm okay and I don't want it to go much further I just quickly get [unclear] this button again and it stops calling out. But when I first got it obviously I knocked it a few times and it calls out. I’d suddenly hear the beeper going on it, oh [unclear]. My son rings from Melbourne, he says are you alright? (P11)*  *the other thing, I've got to take blood pressure readings when I'm not feeling too good or something. I've got to worry about whether my blood pressure is too low or too high. (P11)*  *I leave home here at ten past five and I'm on the [dialysis] machine about twenty to six - quarter to six, depending on how - because I'm the first cab off the rank. (P11)*  *I don't think I ever achieve it [fluid restrictions] over the weekends. My weight's - because they weigh you when you go in and they estimate your body weight and then anything over what they estimate is your body weight is fluid. So that's what they try and take off you. The trouble is, I'm getting a bit healthier, so my body weight is going up and so my fluid level is [laughs]. (P11)*  *I gonna sit in there because I cannot - I can't walk around at all. I would love to walk but my doctor said, you're gonna walk. This Dr Aitken he said, you try to walk. Force yourself. I force myself to walk. (P12)*  *So you do your blood sugar check before you have your insulin, is that right?*  *Male 1: Can I be honest with you, sometimes I don't want to know.*  *Facilitator 1: Okay, sure.*  *Male 1: My wife said, check your sugar how much it is. I said, I don’t want to know. Because my body tells me. (P12)*  *fingers crossed you know they said you know waiting for the big one [heart attack]. I hope the big one never come (P13)*  *First three, first six are the morning pills. Then next six are the evening pills. The bottle is the evening pill. This is in such a way so I never have to go - so what do I do every [evening], I put my doses in there, the morning there, the evening here. So evening dose is there. (P18)*  *as soon as that [weekly medicine pack] finished, every five days when I know it's coming how I do it because sometime when you get up late and you have to run out, I just got it there so I could take it with my breakfast. Then when I come home at night I could take it at night. (P21)*  *I always carry a spare, this is I'm talking about diabetes only, I take a spare tablet, got a tablet with me, so I already have one in my bag. (P21)* |
| 1. Responding to unstable changes in their conditions | *The cardiology told me to take my blood pressure. Three times a day. Yeah, three and I've got to take sitting, standing up, sitting down. Morning, noon, evening. (P24)*  *my eyesight started to go just about the same time as I got the diabetes and my eyesight was going very blurry. It took a long time before we found out whether the blurriness was as a result of the diabetes or whether it was a result of the cataracts. So that took a long time to resolve. (P6)*  *You haven't asked whether I test myself for sugar regularly.*  *Facilitator 2: Oh right. Do you test…*  *Interviewee: No I don't.*  *Facilitator 2: You don't?*  *Interviewee: I used to, but generally a waste of time, because some days it's up a bit. The next day it's all down and it's up and down.*  *Facilitator 2: It fluctuates.*  *Interviewee: That's right. What did it prove? It didn't prove anything anyway. (P16)*  *You were talking about these couple of months that has become worse.*  *Interviewee: Yeah, the last six months or so, yes, yeah.*  *Facilitator 2: Right, and how are you managing it?*  *Interviewee: I haven't got a choice, have I? It's difficult.*  *Facilitator 2: Yeah.*  *Interviewee: As I've said to you, you can't do anything. (P16)*  *I'd like to know and I could stop taking them if I knew, but I don't know.*  *Facilitator 2: Are you generally happy with the medications that you're taking?*  *Interviewee: Oh, I wouldn't say happy, I've got no choice, have I? I want to stay alive for a short while. (P16)* |
| 1. Coming back from crisis before stabilizing | *you have to accommodate and modify your way of thinking every day. Initially, I was very, very upset, not just with myself. Had it been my fault, I would have accepted it a bit better, but because it somebody else's fault (P1)*  *You learn it. As I said, there are restrictions in life and I've learned to try and cope as best as I can. I no longer get frustrated, in the sense, I can't do that. If you can't do it, you just don't do it. I've not driven on freeways or anything, so I don't have any problems. If I need to drive ever again on them, I will see if I can. If I can't, I can't. Public transport for distances (P1)*  *This afternoon I have to go and get that stick that you can use for scratching your back, because you can't - I just cannot reach that part of my back. (P1)*  *It took me, as I said, a good three, maybe four, weeks to get back to accepting that that has happened. At times I felt like going and hitting my head on the wall. I think that is the worst thing that can happen. If I was alone at all in those days, I would have probably been - where, I don't know. There was always somebody with me after I came home from the hospital. (P1)*  *14 hours a day was normal for me to work. Then absolutely nothing. It was very, very hard. In those days, with the medication, I used to sleep for 10, 12 hours during the day. Now it is very difficult for me, and I mean, it is very difficult. (P1)*  *I was so bleary eyed, I put some elastic bands round the end of the banister so that I know if I haven't got my eyes open and I haven't got the phone - we've got the sensor light now, but I'm so bleary in the morning often, so I can feel then, that I've come to the end of the stairs because I nearly fell there.(P5)* |
